# Does Warfarin or Rivaroxaban at Low Anticoagulation Intensity Provide a Survival Benefit to Asian Patients With Atrial Fibrillation?

**DOI:** 10.3389/fcvm.2021.768730

**Published:** 2021-11-25

**Authors:** Dong Lin, Yequn Chen, Jian Yong, Shiwan Wu, Yan Zhou, Weiping Li, Xuerui Tan, Ruisheng Liu

**Affiliations:** ^1^Department of Cardiology, The First Affiliated Hospital of Shantou University Medical College, Shantou, China; ^2^School of Medical and Health Sciences, Edith Cowan University, Perth, WA, Australia; ^3^Clinical Cohort Research Center, The First Affiliated Hospital of Shantou University Medical College, Shantou, China; ^4^Clinical Research Center, The First Affiliated Hospital of Shantou University Medical College, Shantou, China; ^5^Morsani College of Medicine, University of South Florida, Tampa, FL, United States

**Keywords:** atrial fibrillation, warfarin, rivaroxaban, mortality, anticoagulant

## Abstract

**Background:** Low-dose rivaroxaban and low-intensity warfarin are widely used in Asia for patients with atrial fibrillation (AF). However, in Asians, it is unclear whether low-dose rivaroxaban and low-intensity warfarin can improve the prognosis of AF. In this study, we investigate the survival benefits of low-dose rivaroxaban and low-intensity warfarin in Asian patients with AF in clinical practice.

**Methods:** This cohort study used medical records in a single tertiary hospital in China, between 2019 and 2020, to identify patients with AF who used rivaroxaban or warfarin, or had no anticoagulant therapy. Follow-ups were performed through telephone contact or medical record review. Cox proportional hazards models were used to compare the risk of mortality of patients in the anticoagulant-untreated group vs. warfarin-treated groups and rivaroxaban-treated groups.

**Results:** A total of 1727 AF patients, discharged between 2019 and 2020, were enrolled in this cohort, of which 873 patients did not use any anticoagulant, 457 patients received warfarin and 397 patients used rivaroxaban. Multivariable analysis showed that, of all the warfarin groups, patients with an international normalized ratio (INR) below 2, good INR control, or poor INR control had a significantly lower risk of mortality compared with that of patients without anticoagulants (HR 0.309, *p* = 0.0001; HR 0.387, *p* = 0.0238; HR 0.363, *p* < 0.0001). Multivariable Cox proportional hazard analyses also demonstrated that, compared with the no anticoagulant group, all rivaroxaban dosage groups (≤10 mg, HR 0.456, *p* = 0.0129; 15 mg, HR 0.246, *p* = 0.0003; 20 mg, HR 0.264, *p* = 0.0237) were significantly associated with a lower risk of mortality.

**Conclusion:** Despite effects being smaller than observed with recommended optimal anticoagulation, the use of warfarin with an INR below 2, poor INR control and the use of low-dose rivaroxaban may still provide survival benefits, suggesting viable alternatives that enable physicians to better resolve decisional conflicts concerning the risks and benefits of anticoagulant therapies, as well as for patients in need of but unable to receive standard anticoagulant therapy due to bleeding risk or other factors, such as financial burden, concerns of adverse outcomes, as well as low treatment compliance and persistence.

## Introduction

Atrial fibrillation (AF) has a prevalence of 2–3% worldwide (1, 2), with a recent meta-analysis estimating over 7 million adults in China alone having AF (3). AF significantly increases the risk of thromboembolic events, congestive heart failure, and mortality (2, 4). Vitamin K antagonists (primarily warfarin) have been effective antithrombotic therapies for inexpensive prevention of ischemic stroke in AF patients (5). However, warfarin has many issues that limit its use, such as multiple food, drug, and pharmacogenomic interactions, which result in unpredictable pharmacokinetics and pharmacodynamics (5, 6). Since warfarin is associated with risk of bleeding and thromboembolism, warfarin dosing for maintaining anticoagulation therapy must be monitored using the international normalized ratio (INR). As a result, regular coagulation monitoring and dose adjustments are continually required (7). The European Society of Cardiology (ESC) and American Heart Association (AHA) recommend an INR in the range of 2.0–3.0 as a target for the prevention of thromboembolism and hemorrhage in patients with non-valvular AF (8, 9). However, several studies have shown that Asians are more susceptible to warfarin-induced bleeding than non-Asians (10, 11). Previous meta-analyses have shown that in Asian non-valvular AF patients taking warfarin, patients within INR target values of 1.5–2.5 have a reduced risk of bleeding without increases in thromboembolism (12–15). Several studies have found a high proportion of low INR intensity warfarin use in Asians (16), but the efficacy and safety of low INR intensity warfarin use in Asian populations in clinical practice remain unclear.

Direct oral anticoagulants (DOACs), including dabigatran, apixaban, edoxaban, and rivaroxaban (17), are effective and safe alternatives to warfarin for stroke prevention in patients with non-valvular AF (NVAF) (18, 19). Since DOACs do not require routine monitoring of drug concentrations, it is important to select the appropriate dose of DOACs based on the dosing criteria defined in randomized controlled trials. Results from non-Asian and Japanese clinical trials have shown that the efficacy of rivaroxaban is not inferior to warfarin (19, 20). However, populations from randomized controlled trials are usually selected based on strict eligibility criteria and under careful protocol-based follow-up. Therefore, the results of randomized controlled trials may not apply to all patients with AF in clinical settings. Previous studies found that low-dose rivaroxaban (10 mg/day) is widely used in actual clinical practice in Asian countries (21–23). Since Asian populations are associated with a higher risk of bleeding, such as intracranial hemorrhaging (24), physicians tend to prescribe low-dose DOACs for Asian patients with AF in daily clinical practice. However, there is a paucity of evidence on the effects of low-dose rivaroxaban in Asian populations. In this study, we plan to investigate the survival benefits of low-dose rivaroxaban and low-intensity INR warfarin in Asian patients with AF in a clinical setting.

## Methods

### Patients and Data Collection

This was a prospective observational study using data from AF patients who were admitted to the First Affiliated Hospital of Shantou University Medical College from 2019 to 2020. The inclusion criteria were above 18 years of age, had medical conditions that required anticoagulation, received either rivaroxaban, warfarin, or no anticoagulation therapy, and consented to follow-up after the index discharge date. AF patients with the following characteristics were excluded: (1) pregnant; (2) used warfarin with no INR values; (3) used rivaroxaban with missing dosing information; (4) were missing baseline risk factors or demographic information. Patients with evidence of AF from electronic health records or electrophysiologic evaluations were considered as having AF. AF was defined as a supraventricular tachyarrhythmia with uncoordinated atrial electrical activation and consequent ineffective atrial contraction. The electrocardiographic characteristics of AF include irregular R-R intervals (when atrioventricular conduction is not impaired), absence of distinct repeating P-waves, and irregular atrial activations (25). This study was approved by the Ethics Committee of the First Affiliated Hospital of Shantou University Medical College, and all participants provided written informed consent before inclusion in this study.

Subject characteristics at baseline, including medical histories, prior and concomitant medications, demographic characteristics, alcohol, smoking, CHA_2_DS_2_-VASc score, HAS-BLED score, and other clinical characteristics, as well as the usage of treatments and baseline INR, were collected from the medical records. Follow-up INR records, and anticoagulant treatments were procured from telephone visits and the hospital system's outpatient-based electronic medical records. The baseline comorbidities were rheumatic heart disease (RHD), malignancy, chronic kidney disease (CKD), chronic obstructive pulmonary disease diastolic (COPD), thyroid disease, congestive heart failure (CHF), gastrointestinal (GI) bleeding, hypertension, diabetes mellitus, coronary disease, and ischemic stroke. Other clinical characteristics were systolic blood pressure (SBP), diastolic blood pressure (DBP), creatinine (Cr), prothrombin time-international normalized ratio (PT-INR), and ejection fraction (EF). Prior medications were aspirin, clopidogrel, and statin. For quantifying thromboembolic risk, we combined comorbidity information into the CHA_2_DS_2_-VASc score (26), consisting of congestive heart failure, hypertension, age (65–74 years, ≥75 years), diabetes, transient ischemic attack (TIA)/stroke attack, vascular disease, and gender. To assess the risk of bleeding, we calculated the HAS-BLED score (27), which includes hypertension, age > 65, stroke history, renal disease, liver disease, prior major bleeding or predisposition to bleeding, labile INR, medication usage predisposing to bleeding, and alcohol consumption.

Follow-ups were carried out by medical record review and/or telephone interviews at 30, 90, 180, and 365 days after discharge. The follow-up period for patients using rivaroxaban started from the index date of rivaroxaban prescription. The follow-up period of patients without anticoagulants started from the index date of hospital discharge. The follow-up period of patients receiving warfarin began from the last discharge date with warfarin prescription. Patient information, including survival status, INR, and anticoagulant treatment was collected during follow-up through electronic medical records. All INR values were collected during follow-up, independent of the follow-up visits. Patients with missing medical records were contacted via telephone interviews with patients or their family members to collect their information. If patients could not be followed up through electronic medical records or telephone interview, such patients were recorded as lost to follow-up. All patients were followed up to the occurrence of death, switching of treatment (i.e., received OAC for the anticoagulant-untreated group; received an alternative OAC, including dabigatran and apixaban, for the warfarin group and rivaroxaban group), were lost to follow-up, and the end of the study period, whichever came first. For patients who switched anticoagulant treatments during follow-up, only information collected from study enrollment to the day of switching anticoagulant treatments was used for the analysis.

### Study Outcomes

The primary outcome of this study was all-cause mortality, collected using telephone visits and medical records of subjects.

### Statistical Analysis

The warfarin treatment groups were categorized based on the PT-INR (PT-INR <2, 2 ≤ PT-INR ≤ 3, PT-INR>3) collected at the last hospital discharge before the occurrence of death or last contact date. The warfarin treatment groups were also categorized based on the percentage of INR measurements in the therapeutic range (PINRR), with a PINRR ≤ 56.1% regarded as poor INR control and a PINRR > 56.1% regarded as good INR control. PINRR was the number of INR values of 2.0–3.0 of the total number of INR values measured. A cut-off value of PINRR ≤ 56.1% was shown to be a good discriminator of a time to therapeutic range (TTR) <65% (28). Patients using rivaroxaban were categorized into ≤10 mg, 15 mg, and 20 mg groups. In all analyses that used comparisons with the rivaroxaban group, subjects who had moderate to severe mitral stenosis, a bioprosthetic or mechanical prosthetic valve, or end-stage chronic kidney disease with or without dialysis or hypertrophic cardiomyopathy were excluded from the anticoagulant-untreated group.

For continuous variables, data are shown as mean ± standard deviation or median with interquartile ranges. For categorical variables, data are shown as counts with percentages. The baseline characteristics of the anticoagulant-untreated groups were compared with warfarin groups and rivaroxaban groups using the Kruskal-Wallis test for continuous variables and chi-square tests for categorical variables.

Cumulative incidence of mortality was estimated using the Kaplan-Meier method and compared using the log-rank test. Univariable and multivariable Cox proportional hazards models were used to compare the risk of mortality of patients without anticoagulants to patients using warfarin, as well as patients receiving rivaroxaban. Hazard ratios (HRs) with 95% confidence intervals (CIs) are presented. Multivariable models for the comparison of patients using warfarin and patients without anticoagulant medication was adjusted for gender, age, TIA/stroke, malignancy, CKD, and GI bleeding. Multivariable models for the comparison of patients receiving rivaroxaban and patients without anticoagulants were adjusted for gender, age, coronary disease, TIA/stroke, aspirin, CHF, malignancy, CKD, and statin. Statistical analyses were performed using SPSS version 22.0 (SPSS Inc., Chicago, Illinois, USA), and figures were constructed using R software version 3.6.1 (R Foundation for Statistical Computing, Vienna, Austria). Two-tailed *p* < 0.05 were considered statistically significant.

## Results

### Baseline Characteristics

A total of 2092 patients with AF, who were discharged between 2019 and 2020, were enrolled in this cohort. Among the 2092 participants, 204 patients were excluded because of lack of follow-up data, 49 patients in the warfarin-treated group were excluded because of missing INR values, 86 patients in the rivaroxaban-treated group were excluded because of missing dosing information, and 26 patients were excluded owing to lack of baseline risk factors or demographic information. The final analysis included 1,727 patients with diagnosed AF, of which 873 patients did not use any anticoagulant, 457 patients received warfarin and 397 patients used rivaroxaban. Baseline characteristics of patients without anticoagulants, patients using warfarin (PT-INR < 2, 2 ≤ PT-INR ≤ 3, PT-INR > 3, PINRR > 56.1%, PINRR ≤ 56.1%), and NVAF patients receiving rivaroxaban (≤ 10, 15, 20 mg) are shown in Tables 1A,B, respectively. In addition to age, CHA_2_DS_2_-VASc score, and HAS-BLED score, comorbidities (congestive heart failure, hypertension, coronary disease), smoking, biomarkers (systolic blood pressure, creatinine, and PT-INR), use of other medications (statin, aspirin, or clopidogrel) were significantly different between patients not using anticoagulants and either patients using warfarin or patients receiving rivaroxaban. Gender was significantly different in the comparison of patients without anticoagulants with patients receiving warfarin but not with patients using rivaroxaban. Chronic kidney disease, diabetes, GI bleeding, and ischemic stroke were significantly different in the comparison of patients without anticoagulants with patients receiving rivaroxaban.

**Table 1:** Baseline characteristics of enrolled patients (A) warfarin and (B) rivaroxaban.

**Characteristics**	**No anticoagulant (*n* = 873)**	**PT-INR**				**PINRR**		
		**PT-INR <2 (*n* = 330)**	**PT-INR 2–3 (*n* = 93)**	**PT-INR > 3 (*n* = 34)**	***P***	**≤56.1% (*n* = 354)**	**>56.1% (*n* = 103)**	***P***
**(A) WARFARIN**								
Gender (female)	345 (39.5%)	179 (54.2%)	41 (44.1%)	19 (55.9%)	<0.001	187 (52.8%)	52 (50.5%)	<0.001
Age (years)	70.45 ± 12.61	64.16 ± 11.45	64.75 ± 10.56	67.15 ± 7.97	<0.001	64.42 ± 11.30	64.51 ± 9.87	<0.001
CHA_2_DS_2_-VASc score	3.48 ± 1.85	3.06 ± 1.68	2.95 ± 1.39	3.24 ± 1.5	<0.001	2.85 ± 1.64	2.92 ± 1.49	<0.001
HAS-BLED score	2.7 ± 1.19	2.34 ± 1.19	2.36 ± 1.14	2.61 ± 1.14	<0.001	2.28 ± 1.08	2.45 ± 1.20	<0.001
RHD	34 (3.95%)	100 (30.9%)	38 (41.3%)	11 (34.4%)	<0.001	105 (29.7%)	44 (42.7%)	<0.001
CKD	122 (14.2%)	33 (10.2%)	10 (10.9%)	7 (21.9%)	0.11	39 (11.0%)	11 (10.7%)	0.49
COPD	64 (7.4%)	10 (3.1%)	4 (4.4%)	1 (3.1%)	0.031	11 (3.1%)	4 (3.9%)	0.0358
CHF	401 (45.9%)	225 (68.2%)	70 (75.3%)	24 (70.6%)	<0.001	245 (6.9%)	74 (7.2%)	<0.001
GI bleeding	32 (3.7%)	4 (1.2%)	1 (1.1%)	2 (6.3%)	0.06	6 (1.7%)	1 (1.0%)	0.14
Hypertension	541 (62.9%)	149 (46.0%)	37 (40.2%)	13 (40.6%)	<0.001	157 (44.4%)	42 (40.8%)	<0.001
Diabetes mellitus	238 (27.7%)	79 (24.9%)	24 (26.1%)	12 (37.5%)	0.37	89 (25.1%)	26 (25.2%)	0.75
Coronary disease	326 (37.9%)	77 (23.8%)	20 (21.7%)	4 (12.5%)	<0.001	84 (23.7%)	17 (16.5%)	<0.001
Ischemic stroke	228 (26.5%)	72 (22.2%)	16 (17.4%)	4 (12.5%)	0.05	74 (20.9%)	18 (17.5%)	0.095
Smoking	270 (30.9%)	70 (21.2%)	23 (24.7%)	5 (14.7%)	0.002	76 (21.5%)	22 (21.4%)	0.0173
SBP (mmHg)	139.6 ± 47.0	131.6 (23.1)	128.5 ± 24.8	130.4 ± 24.2	0.032	131.8 ± 22.7	129.3 ± 22.0	<0.001
DBP (mmHg)	86.29 ± 41.0	84 ± 18.1	79.45 ± 15.2	84.17 ± 12.7	0.53	83.5 ± 17.1	81.1 ± 14.2	0.17
Cr (μmol/L)	126.96 ± 91.1	118.07 ± 98.0	115.36 ± 57.1	127.42 ± 42.6	<0.001	116.4 ± 81.3	116.6 ± 52.0	0.23
PT-INR	1.16 ± 0.54	1.5 ± 1.31	2.22 ± 0.95	3.79 ± 0.95	0.013	1.61 ± 1.46	2.11 ± 0.94	<0.001
EF (%)	58.01 ± 12.2	57.71 ± 12.6	58.17 ± 12.6	59.31 ± 11.8	0.19	57.84 ± 11.07	57.47 ± 9.84	0.61
Aspirin-clopidogrel	467(53.5%)	95(28.8%)	13(14.0%)	4 (11.8%)	<0.001	98 (27.7%)	14 (13.6%)	<0.001
Statin	489 (56.0%)	158 (47.9%)	39 (41.9%)	10 (29.4%)	<0.001	166 (46.9%)	41 (39.8%)	0.0021
**Characteristics**	**No anticoagulant** **(*****n*** **=** **813)**	**Rivaroxaban**			
		**≤10 mg** **(*****n*** **=** **131)**	**15 mg** **(*****n*** **=** **169)**	**20 mg** **(*****n*** **=** **97)**	***P***
**(B) RIVAROXABAN**					
Sex (Female)	316 (38.9%)	58 (42.3%)	82 (42.9%)	30 (28.0%)	0.0276
Age (years)	70.70 ± 12.41	74.74 ± 9.0	71.12 ± 9.80	62.06 ± 10.30	<0.001
CHA2DS2-VASc score	3.43 ± 1.83	4.22 ± 1.50	3.35 ± 1.64	2.09 ± 1.60	<0.001
HAS-BLED score	2.66 ± 1.16	2.91 ± 1.12	2.45 ± 1.05	1.8 ± 1.11	<0.001
RHD	8 (0.98%)	3 (2.2%)	5 (2.6%)	2 (1.9%)	0.37
CKD	98 (12.05%)	21 (15.7%)	16 (8.4%)	7 (6.7%)	0.0302
COPD	56 (6.89%)	11 (8.2%)	18 (9.4%)	3 (2.9%)	0.20
CHF	308 (37.9%)	81 (59.1%)	99 (51.8%)	26 (24.3%)	0.0017
GI bleeding	25 (3.08%)	6 (4.5%)	1 (0.5%)	0 (0)	0.081
Hypertension	505 (62.1%)	100 (74.6%)	125 (65.5%)	55 (52.4%)	<0.001
Diabetes mellitus	230 (28.3%)	46 (34.3%)	43 (22.5%)	20 (19.1%)	0.0076
Coronary disease	308 (37.9%)	65 (48.5%)	84 (44.0%)	36 (34.3%)	0.0017
Ischemic stroke	219 (26.9%)	44 (32.8%)	38 (19.9%)	17 (16.2%)	<0.001
Smoking	257 (31.6%)	29 (21.2%)	45 (23.6%)	37 (34.6%)	0.0084
SBP (mmHg)	138.86 ± 46.3	139.6 ± 21.1	138.1 ± 24.0	130.7 ± 20.6	<0.001
DBP (mmHg)	85.97 ± 39.95	84.3 ± 14.4	87.0 ± 16.5	83.2 ± 12.1	0.25
Cr (μmol/L)	118.89 ± 61.56	119.2 ± 43.3	105.4 ± 28.3	104.2 ± 25.1	0.072
PT-INR	1.18 ± 0.46	1.13 ± 0.31	1.14 ± 0.71	1.07 ± 0.24	0.41
EF (%)	58.1 ± 9.98	57.51 ± 13.2%	58.36 ± 12.28	61.46 ± 10.14	0.27
Aspirin-clopidogrel	444 (54.6%)	72 (52.6%)	80 (41.9%)	31 (29.0%)	<0.001
Beta-blocker	484 (59.5%)	73 (54.9%)	106 (64.5%)	66 (70.2%)	0.097
Statin	476 (58.6%)	101 (73.7%)	110 (57.6%)	54 (50.5%)	<0.001

*Data are number (%), median (interquartile 1, interquartile 3) or mean ± SD; PT-INR, prothrombin time international normalized ratio; PINRR, the percentage of INR measurements in range; CHA_2_DS_2_-VASc score includes congestive heart failure, hypertension, age (65–74 years, ≥75 years), diabetes, stroke/transient ischemic attack, vascular disease, sex; HAS-BLED, hypertension, abnormal liver/renal function, stroke history, bleeding history or predisposition, labile INR, elderly, drug/alcohol usage; RHD, rheumatic heart disease; COPD, chronic obstructive pulmonary disease; CHF, congestive heart failure; GI, gastrointestinal; ICH, intracranial hemorrhage; SBP, systolic blood pressure; DBP, diastolic blood pressure; Cr, creatinine; EF, ejection fraction*.

### Comparison Between Warfarin- vs. Anticoagulant-Untreated Groups for Mortality

**Figure 1:**
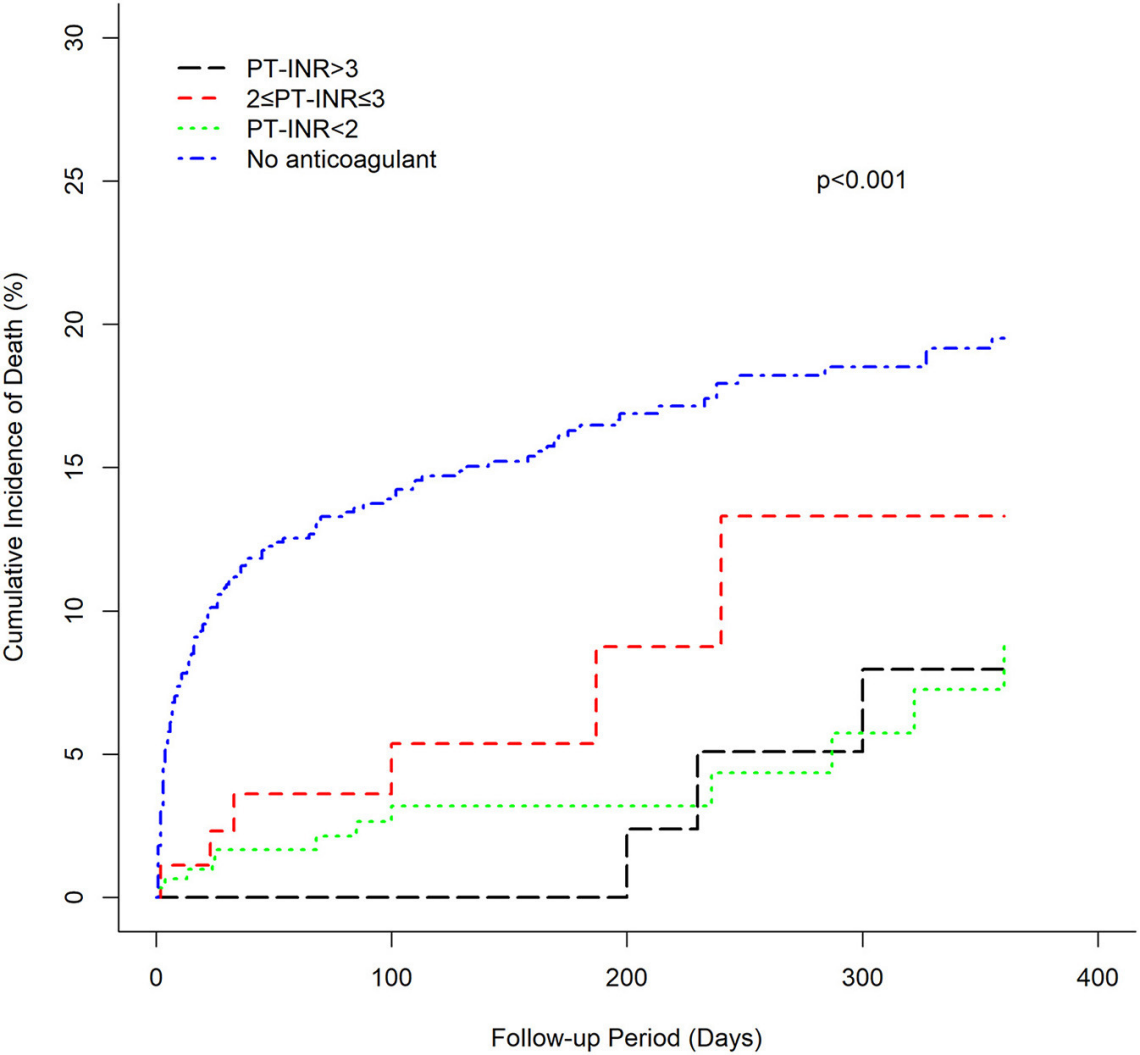
Cumulative mortality of warfarin groups and the anticoagulant-untreated group (PT-INR).

**Figure 2:**
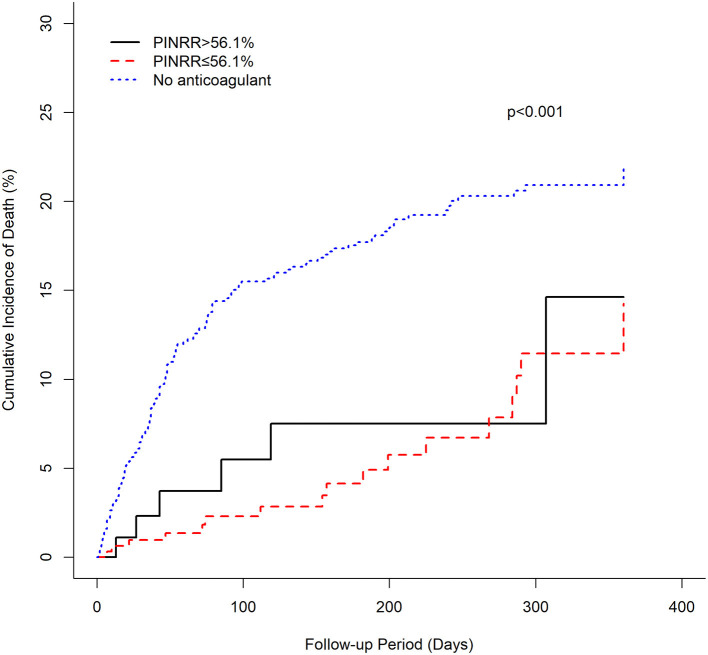
Cumulative mortality of warfarin groups and the anticoagulant-untreated group (PINRR).

The cumulative incidence of mortality in patients using warfarin (PT-INR < 2, 2 ≤ PT-INR ≤ 3, PT-INR > 3), as well as PINRR (>56.1%, ≤ 56.1%), was lower than that of patients not using anticoagulants (log-rank *p* < 0.001) (Figures 1, 2). To delineate the associations between warfarin usage and the risk of mortality, Cox proportional hazards models were constructed (Table 2). Univariate analysis revealed that patients with a PT-INR <2 had only 0.274 times the risk of mortality compared with patients not using anticoagulants. After adjustment for gender, age, TIA/stroke, malignancy, chronic kidney disease, and GI bleeding, patients with a PT-INR < 2 were significantly associated with a lower risk of mortality (HR 0.309, 95%CI 0.170–0.56, *p* = 0.0001) (Table 2). Compared with the no anticoagulant group, both PINRR ≤ 56.1% and PINRR > 56.1% groups had significantly lower risk of mortality in univariate and multivariable analysis (Table 2).

**Table 2:** Univariate and multivariable analysis comparing the mortality of patients receiving warfarin vs. no anticoagulant treatment.

	**Unadjusted**		**Adjusted**	
	**HR (95% CI)**	***P***	**HR (95% CI)**	***P***
**No anticoagulant**	Reference		Reference	
**PT-INR**				
<2	0.274 (0.158, 0.475)	<0.0001	0.309 (0.170, 0.560)	0.0001
2–3	0.444 (0.196, 1.007)	0.052	0.539 (0.236, 1.229)	0.14
>3	0.571 (0.182, 1.794)	0.34	0.652 (0.207, 2.052)	0.46
PINRR^*^				
≤ 56.1%	0.345 (0.211, 0.562)	<0.0001	0.363 (0.220, 0.599)	<0.0001
>56.1%	0.428 (0.189, 0.969)	0.0418	0.387 (0.170, 0.882)	0.0238

*HR, hazard ratio; CI, confidence interval; PT-INR, prothrombin time international normalized ratio; PINRR, the percentage of INR measurements in range; p <0.05 statistically significant; PT-INR vs. no anticoagulant was adjusted for gender, age, TIA/stroke, malignancy, CKD, and GI bleeding*.

* *PINRR vs. no anticoagulant was adjusted for age, coronary disease, left ventricular heart failure, malignancy, and aspirin*.

### Comparison Between Rivaroxaban- vs. Anticoagulant-Untreated Groups for Mortality

**Figure 3:**
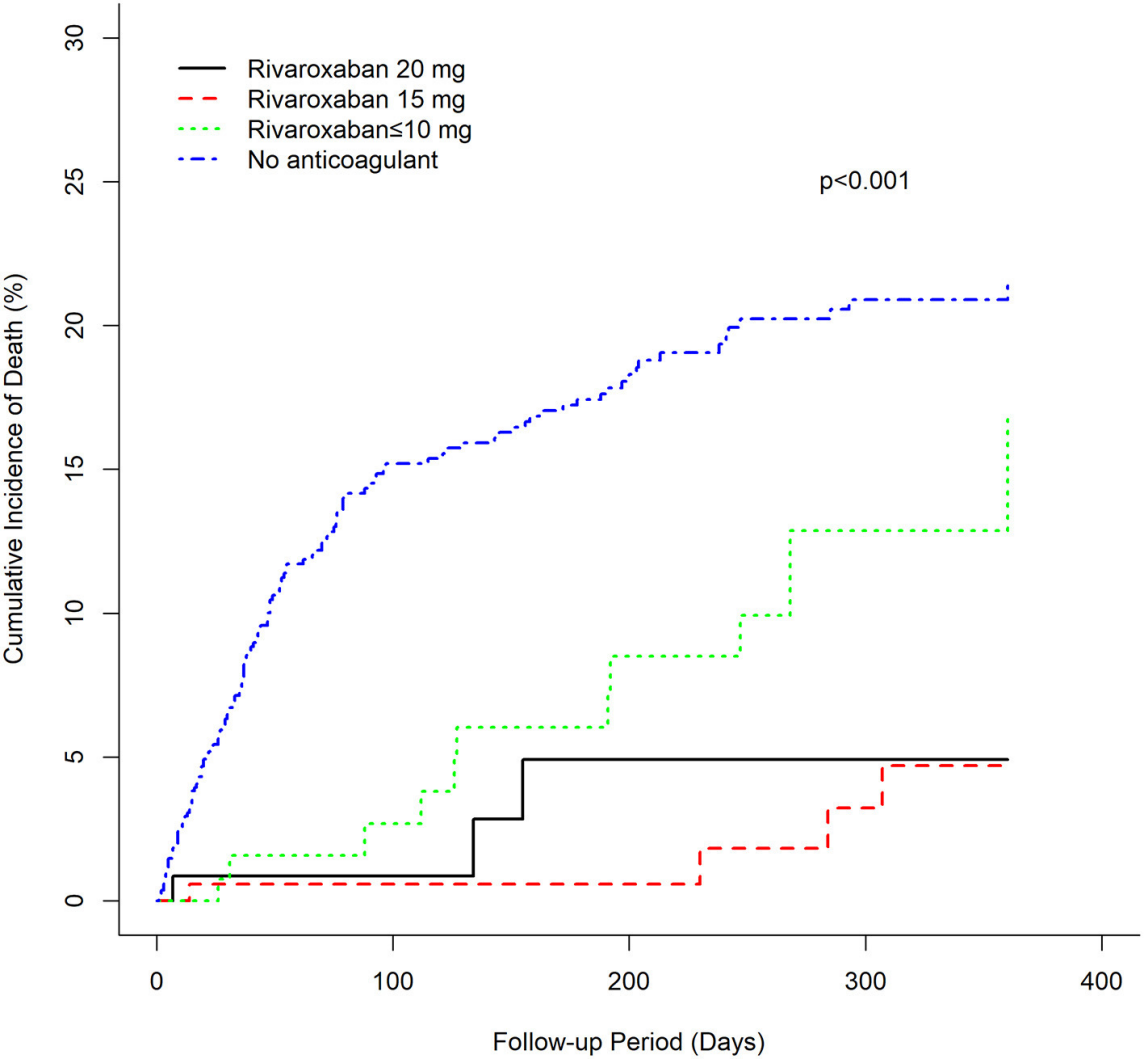
Cumulative mortality of rivaroxaban groups and the anticoagulant-untreated group.

There was a clear trend showing that NVAF patients using rivaroxaban (≤ 10, 15, 20 mg) had a significantly lower incidence of mortality than patients without anticoagulant treatment (log-rank test *p* < 0.001) (Figure 3). Similarly, univariate and multivariable Cox proportional hazard model analyses also demonstrated that, compared with the anticoagulant-untreated group, rivaroxaban treatment at all dosages (≤10 mg, HR 0.454, 95%CI 0.256–0.804, *p* = 0.0068; 15 mg, HR 0.139, 95%CI 0.051–0.376, *p* = 0.0001; 20 mg, HR 0.276, 95%CI 0.087–0.874, *p* = 0.0286) was significantly associated with lower risk of mortality in NVAF patients (Table 3).

**Table 3:** Univariate and multivariable analysis comparing the mortality of patients receiving rivaroxaban vs. no anticoagulant treatment.

	**Unadjusted**		**Adjusted**	
	**HR (95% CI)**	***P***	**HR (95% CI)**	***P***
No anticoagulant	Reference		Reference	
Rivaroxaban ≤ 10 mg	0.529 (0.300, 0.935)	0.0285	0.454 (0.256, 0.804)	0.0068
Rivaroxaban 15 mg	0.136 (0.050, 0.368)	<0.0001	0.139 (0.051, 0.376)	0.0001
Rivaroxaban 20 mg	0.171 (0.055, 0.538)	0.0025	0.276 (0.087, 0.874)	0.0286

*HR, hazard ratio; CI, confidence interval; p < 0.05 statistically significant; Adjusted for age, gender, coronary disease, TIA/stroke, CHF, CKD, aspirin, and statin*.

## Discussion

In this study, we investigated the risk of mortality in Asians with AF who received warfarin, rivaroxaban, or no anticoagulant therapy. The main findings of this study are as follows: (1) among patients taking warfarin, patients with an INR lower than 2, as well as PINRR ≤ 56.1% and PINRR > 56.1%, had a significantly lower risk of mortality than that of patients without anticoagulant therapy, suggesting that warfarin still noticeably reduces the risk of mortality in Asians despite not achieving a target standard-intensity INR of 2–3; (2) low-dose rivaroxaban treatment (10 mg/day) is associated with a significantly lower risk of mortality than in patients not treated with anticoagulants, indicating that low-dose rivaroxaban may have survival benefits for Asians.

Previous studies have found a high percentage of AF patients do not follow the practice guidelines, especially in the Asian population (2, 3). Compared with Caucasians, Asians have a higher incidence of massive hemorrhaging, including intracranial hemorrhaging (10, 29, 30). Therefore, many physicians and patients in Asia are concerned about warfarin-induced bleeding and thrombosis, resulting in low-intensity INR warfarin becoming common in Asia (31–33). Our study shows that low-intensity INR warfarin substantially reduces mortality. A recent meta-analysis of 32 randomized controlled trials in East Asia showed no significant differences in the mortality of AF patients using warfarin with lower INR and that of AF patients using warfarin with standard INR. These findings show that the use of warfarin provides a survival benefit even in cases with a low INR target, suggesting that the use of warfarin with a low INR target might be beneficial to Asian patients who prefer warfarin but cannot have their anticoagulation intensity frequently monitored.

Intake of direct oral anticoagulants for the prevention of stroke in patients with AF is increasing rapidly worldwide, but varies widely, depending mainly but not exclusively on the socioeconomic status of the country under consideration (34, 35). Since DOACs do not require routine monitoring of drug concentrations, it is important to use an appropriate dose of DOAC based on the dosing criteria defined in a randomized controlled trial. However, the prescription of off-label DOACs remains a major problem in daily practice (36). One of the possible reasons is the concern of drug-induced adverse outcomes. Our study shows that various dosages of rivaroxaban, including 10 mg or less, can substantially reduce mortality, providing evidence of benefits associated with low-dose rivaroxaban use. This finding suggests that the use of low-dose rivaroxaban may be considered as an alternative, or in preference to no anticoagulant therapy, especially in situations where physicians have decisional conflicts with the risks and benefits of rivaroxaban.

Furthermore, several studies have shown that DOACs are more commonly studied in low-risk populations (37). Advanced age, frailty, and co-morbidities are common characteristics of the elderly population, making them less likely to be treated with DOACs (38). In our study, the mean age of the low-dose rivaroxaban group (74.7 years old) was ~4 years older than that of the anticoagulant-untreated group (70.7 years old). Although there were larger proportions of comorbidities and higher CHA_2_DS_2_-VASc and HAS-BLED scores in the low-dose rivaroxaban group compared to that of the anticoagulant-untreated therapy group, the risk of mortality was still significantly lower in the low-dose rivaroxaban group. This result indicates that rivaroxaban may substantially reduce the risk of mortality in Asian patients with AF, suggesting that the use of low-dose rivaroxaban may be an alternative for patients, especially elderly patients, who have concerns about the adverse effects and costs of standard-dose rivaroxaban.

### Strengths and Limitations

Due to the complex clinical profile of patients with AF, certain patients were usually excluded from previous randomized controlled trials, making it challenging for physicians to prescribe anticoagulation therapy in clinical practice. On the one hand, our study includes a wide range of AF patients encountered in clinical practice. Therefore, our study could, to some extent, reflect the treatment patterns and associated risks of mortality in the Chinese population. On the other hand, our study observes treatment patterns and outcomes after discharge, and as such, it examines the associations of post-hospitalization anticoagulant dosage use patterns and death. The drug dose of OACs was as per the attending physician's discretion based on the patients' conditions and was collected from the medical records. Non-compliance with guidelines for warfarin and rivaroxaban use may be influenced by many factors. First, the perceived risks of bleeding impeded clinicians from prescribing anticoagulants and patients to adhere to therapy. Second, some patients with low body weight and/or renal impairment were underdosed because of a fear of toxicity. Finally, the high cost of rivaroxaban limited the options for some patients in the cohort. Nevertheless, there are limitations to this study. First, our patients are from a hospital that may have introduced selection bias. Second, our study does not include clinical information, such as bleeding events, thrombosis events, and BMI because of unclear or incomplete data. Third, although TTR has been recommended by the main international guidelines (39, 40) as a measure of the quality of anticoagulation control, it has not gained much popularity in clinical practice due to its tedious calculation; PINNR is much easier to obtain and simpler to calculate in everyday clinical practice, and was shown to have a good correlation with TTR (28). Finally, our sample size is relatively small, and is especially small for subjects with INR 2–3, so this study might have insufficient power to achieve statistical significance in the analyses for subjects with INR 2–3.

## Conclusion

In Asian patients with AF, the risk of death is significantly lower in both patients receiving rivaroxaban and patients using warfarin with an INR below 2 in comparison with patients without anticoagulant therapy. These findings show that, despite effects being smaller than obtained with recommended doses, the use of warfarin below the standard INR target and the use of low-dose rivaroxaban still provide survival benefits, suggesting viable alternatives to physicians to better resolve decisional conflicts with the risks and benefits of anticoagulant therapy, as well as to patients in need of anticoagulant therapy but are not receiving it due to bleeding risk or other factors, such as financial burden, concerns of adverse outcomes, as well as low treatment compliance and persistence.

## Data Availability Statement

The raw data supporting the conclusions of this article will be made available by the authors, without undue reservation.

## Ethics Statement

The studies involving human participants were reviewed and approved by Ethics Committee of the First Affiliated Hospital of Shantou University Medical College. The patients/participants provided their written informed consent to participate in this study.

## Author Contributions

XT and YC: contributed to the conception and design of the work. DL, JY, SW, YZ, and WL: contributed to the data collection and data management. YC and DL: contributed to the analysis, interpretation of data, and drafted the manuscript. XT, RL, and YC: reviewed and edited the manuscript. All authors contributed to the article and approved the submitted version.

## Funding

This study was supported by projects from Grant for Key Disciplinary Project of Clinical Medicine under the High-level University Development Program (2020), Innovation Team Project of Guangdong Universities (2019KCXTD003), Li Ka Shing Foundation Cross-Disciplinary Research Grant (2020LKSFG19B), Funding for Guangdong Medical Leading Talent (2019–2022), National Natural Science Foundation of China (82073659), and Dengfeng Project for the construction of high-level hospitals in Guangdong Province—the First Affiliated Hospital of Shantou University Medical College (202003-2).

## Conflict of Interest

The authors declare that the research was conducted in the absence of any commercial or financial relationships that could be construed as a potential conflict of interest.

## Publisher's Note

All claims expressed in this article are solely those of the authors and do not necessarily represent those of their affiliated organizations, or those of the publisher, the editors and the reviewers. Any product that may be evaluated in this article, or claim that may be made by its manufacturer, is not guaranteed or endorsed by the publisher.
